# A simplified approach to control cell adherence on biologically derived in vitro cell culture scaffolds by direct UV-mediated RGD linkage

**DOI:** 10.1007/s10856-020-06446-x

**Published:** 2020-10-14

**Authors:** A. M. Porras Hernández, H. Pohlit, F. Sjögren, L. Shi, D. Ossipov, M. Antfolk, M. Tenje

**Affiliations:** 1 Science for Life Laboratory, Department of Materials Science and Engineering, Uppsala University, Uppsala, Sweden; 2 Department of Chemistry-Ångström, Uppsala University, Uppsala, Sweden; 3 Department of Biosciences and Nutrition (BioNut), Karolinska Institutet, Huddinge, Sweden; 4 BRIC—Biotech Research and Innovation Centre, Faculty of Health and Medical Sciences, University of Copenhagen, Copenhagen, Denmark; 5 Novo Nordisk Foundation Center for Stem Cell Research, Faculty of Health and Medical Sciences, University of Copenhagen, Copenhagen, Denmark

## Abstract

In this work, we present a method to fabricate a hyaluronic acid (HA) hydrogel with spatially controlled cell-adhesion properties based on photo-polymerisation cross-linking and functionalization. The approach utilises the same reaction pathway for both steps meaning that it is user-friendly and allows for adaptation at any stage during the fabrication process. Moreover, the process does not require any additional cross-linkers. The hydrogel is formed by UV-initiated radical addition reaction between acrylamide (Am) groups on the HA backbone. Cell adhesion is modulated by functionalising the adhesion peptide sequence arginine–glycine–aspartate onto the hydrogel surface via radical mediated thiol–ene reaction using the non-reacted Am groups. We show that 10 × 10 µm^2^ squares could be patterned with sharp features and a good resolution. The smallest area that could be patterned resulting in good cell adhesion was 25 × 25 µm^2^ squares, showing single-cell adhesion. Mouse brain endothelial cells adhered and remained in culture for up to 7 days on 100 × 100 µm^2^ square patterns. We see potential for this material combination for future use in novel organ-on-chip models and tissue engineering where the location of the cells is of importance and to further study endothelial cell biology.

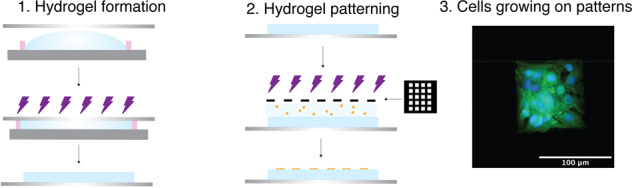

## Introduction

Biomaterials are widely used in biological research and pharmaceutical development as biomimetic cell culture scaffolds to increase the in vivo resemblance of in vitro models [1]. For this purpose, hydrogels belong to a particularly attractive group of biomaterials due to their high-water content, which mimics the in vivo extracellular matrix (ECM) mechanical and physical properties. Furthermore, their high permeability for oxygen and nutrients [2] is important to support long-term cell cultures. In most situations, cell adhesion on the whole hydrogel scaffold is preferred, but for some applications, such as cell–cell interaction studies, one may wish to control the cell adhesion spatially. This can be achieved by preparing a non-adhesive hydrogel and patterning cell-adhesion motifs in the areas of interest. Previous reports have achieved this using synthetic hydrogels such as poly(ethylene glycol)-diacrylate where controlled cell adhesion was induced via the peptide sequence Arg–Gly–Asp (RGD) [3, 4]; poly(vinyl alcohol) using polydopamine to achieve cell adhesion [5] and on polyacrylamide using fibronectin and laminin to control the adhesion of the cells [6]. While synthetic and inert materials offer greater control over the biological responses and material properties, they do lack the inherent biological activity that naturally derived hydrogels hold [2, 7, 8].

For studies of cell interactions in the neurovascular unit, hyaluronic acid (HA), a naturally derived polysaccharide, represents a particular interest as it constitutes an integral part of the brain ECM [9]. HA is a glycosaminoglycan and the high molecular weight HA does not promote cell adhesion, which lends itself as an ideal substrate for controlled cell–cell interaction studies. In previous work using HA, cell adhesion was achieved by linking RGD peptides to the HA molecule by Michael-type addition reactions prior to hydrogel formation [10–15]. However, these approaches do not allow for selective functionalization of adhesion peptides in spatially defined areas due to the ubiquitous presence of peptide throughout the complete 3D structure of the hydrogel. Attachment of adhesion peptides after the hydrogel formation would enable a greater control over the scaffold fabrication process. This is made possible using photo-induced radical addition reactions in combination with photomasks shielding some areas of the hydrogel and following this approach, radical thiol–ene reactions, one type of UV-initiated radical addition reactions, have been used [16–18]. Jing et al. [19] used this reaction to both form the hydrogel and attach the RGD peptide, but as the RGD peptide is attached to the HA chains before hydrogel formation, spatial control of the functionalised patterns could still not be obtained. In a previous publication, we immobilised RGD by radical thiol–ene addition in a 3D HA-acrylamide (HA-am) hydrogel with spatial control using additive manufacturing but we did not investigate cell adhesion [16]. Gramlich et al. [17] used radical thiol–ene addition to form the gel and attach peptide from norbornylated HA and a di-thiol cross-linker. Later, the same group patterned RGD peptides using radical thiol–ene addition after forming electrospun HA scaffolds by Michael-type addition reaction [18]. Griffin et al. [20] used a more sophisticated two-step process that includes a UV-mediated deprotection of the reaction site for RGD binding after hydrogel formation by Michael-type addition reaction [20]. Goubko et al. [21] formed the hydrogel and attached “caged” RGD peptides via amidation reactions where uncaging was spatially controlled using photolabile caging groups. All these methods do show controlled cell adhesion but they include either the necessity of a di-thiol cross-linker molecule, complex sample preparation processes (electrospinning) or multiple time-consuming steps to bind the RGD peptides (using protection groups, “caging”).

In this work, we show a simplified approach using moulded culture scaffolds and a direct UV-mediated RGD linking approach. To make the process as user-friendly and flexible as possible, we have prepared an HA derivative (HA-am) where the acrylamide (Am) groups of the HA-am macromolecules serve both as the cross-linking and the functionalization groups via UV-induced radical addition reaction. This eliminates the need for the addition of cross-linker molecules like di-thiols, which might alter the material properties of the final hydrogel scaffold. Both cross-linking and peptide attachment are initiated by UV light exposure enabling fast reaction kinetics and spatio-temporal control [22]. In this work, we show that micrometre scale features of RGD could be patterned on the hydrogel, down to 10 × 10 µm^2^, and that these patterns result in spatial control of mouse brain microvascular endothelial cell adhesion.

## Materials and methods

Sodium hyaluronate (100–150 kDa) was purchased from Lifecore Biomedical. Dialysis membranes Spectra/Por (MW cut off 3.5 kDa) were purchased from SpectrumLabs. Tetrahydrofuran dry, hydrochloric acid (HCl) 37%, Dulbelco’s phosphate buffer saline (DPBS) and sodium chloride were purchased from Fischer Scientific. 3-(Trimethoxysilyl)propyl methacrylate, *N*-Boc-ethylenediamine, triethylamine anhydrous, acryloyl chloride, dioxane, *N*-(3-dimethylaminopropyl)-*N*′-ethylcarbodiimide hydrochloride, 1-hydroxybenzotriazole hydrate, acetonitrile anhydrous, sodium hydrogen carbonate and photo-initiator Irgacure 2959 were purchased from Sigma Aldrich. Calcein AM, propidium iodide, minimum essential medium (MEM) without phenol red, Dulbecco’s modified Eagle medium (DMEM), high glucose Glutamax and fetal bovine serum (FBS) were purchased from Fisher Scientific. RGDSC, 5FAM-RGDSC, GCGYRGDSPG and 5FAM-GCGYRGDSPG peptides were purchased from Innovagen AB. Glass coverslips and absolute ethanol were purchased from VWR. Plus One Repel-Silane ES was purchased from GE Healthcare Life Sciences.

### Synthesis of *N*-(2-aminoethyl) acrylamide linker

**Fig. 1:**
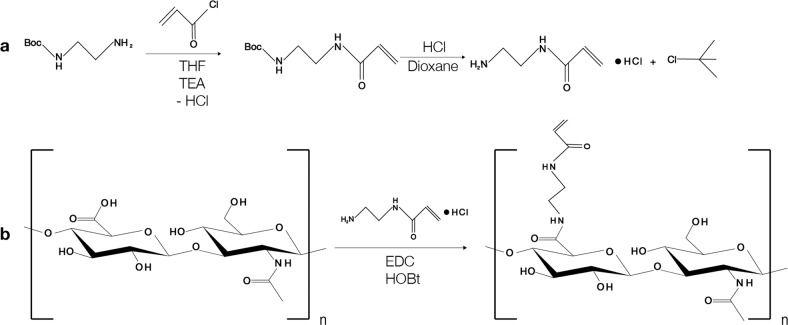
**A** Synthesis of *N*-(2-aminoethyl) acrylamide linker and **B** subsequent preparation of HA-am derivative by amidation of native HA with the linker

*N*-(2-aminoethyl) acrylamide linker was prepared by a three-step synthesis as described previously [23]. Briefly, *N*-Boc-ethylenediamine (4.0 g, 25 mmol) was diluted in dry tetrahydrofuran. Triethylamine (3.0 g, 30 mmol) was added to the solution and cooled down in an ice bath. In a separate flask, 2.44 mL (30 mmol) solution of acryloyl chloride in dry THF was prepared and added dropwise to the *N*-Boc-ethylenediamine (4.0 g, 25 mmol) over 6 min. The reaction mixture was left magnetically stirred on an ice bath for 2 h and afterwards at room temperature overnight. The mixture was then diluted with a solution of sodium hydrogen carbonate (NaHCO_3_) and finally mixed with dichloromethane (DCM). The two-phase mixture was separated, the DCM phase was washed with NaHCO_3_ (100 mL) and saturated brine (100 mL). The product, *N*-Boc-aminoethyl acrylamide (Boc-AEAA), was isolated by drying the DCM phase over sodium sulphate and finally evaporating the solvent on a rotary evaporator. The residue was dried in a vacuum chamber overnight. To remove the Boc protecting group, the Boc-AEAA was diluted with a solution containing concentrated HCl and dioxane (Fig. 1A). The mixture was stirred for 4 h, protected from light and at room temperature and finally evaporated.

### Synthesis of hyaluronic acid-acrylamide derivative (HA-am)

HA was functionalized with Am groups by reacting the carboxylic acid group of the HA backbone and the amino group of *N*-(2-aminoethyl) acrylamide linker (Fig. 1B) according to previous reports [24]. Sodium hyaluronate (200 mg, MW = 135 kDa) was dissolved in deionized water at a concentration of 8 mg/mL. *N*-(2-aminoethyl) acrylamide linker (56 mg) was added to the HA solution. HOBt (72 mg) was separately dissolved in a 1:1 (v/v) mixture acetonitrile (1.4 mL) and deionized H_2_O (1.4 mL) at a concentration 27 mg/mL (3.0 mL) by slight heating. Once the HOBt solution was cooled to room temperature, it was added to the HA solution. The pH of the reaction solution was adjusted to pH 6 with 1 M NaOH. The coupling reaction was initiated by the addition of EDC (155 mg). The reaction was stirred at room temperature overnight in the dark. The reaction solution was then transferred to a dialyzing membrane and dialysed against NaCl solution (0.1 M) adjusted to pH 3.5 with 1 M HCl for 24 h. Subsequently, the dialysis solvent was exchanged twice to distilled water adjusted to pH 3.5 and twice to neutral distilled water. The solution was filtered to give rise to a clear and transparent solution. The filtered solution was freeze-dried to give a white paper-like material. The degree of modification with Am groups was calculated by ^1^H NMR in D_2_O, indicating that 14% of HA disaccharide repeating units were modified with Am groups.

### Fabrication of moulds for hydrogel formation

Moulds for preparation of the hydrogel samples for the cell studies were fabricated using standard lithography techniques. Dry film photoresist (SUEX film) 200 µm thick (DJ Microlaminates Inc) were laminated to 4″ silicon wafer using a benchtop laminator. The SUEX film was exposed to UV light (30 s, eight cycles) using a mask aligner (KarlSüss M6) equipped with a 350 W Hg lamp (365 nm). The film was exposed through a transparency photomask consisting of 200-µm-wide rings with a 7.8-mm-inner diameter. After exposure, the SUEX film was baked at 85 °C for 1 h and the pattern was developed for 40 min in developer mr-Dev500 (Micro Resist Technology GmbH) and subsequently hard baked for 1 h at 200 °C. There were in total 30 moulds that were diced for individual use with a dicing saw (Disco DAD 361). The final mould height (~220 µm) was determined using an optical profilometer (ZYGO). The moulds were treated with Repel-One-Silane (GE Healthcare) following the supplier’s instructions to reduce the hydrogel adhesion to the moulds.

For the rheology samples another mould was prepared comprising three layers of 500 µm PDMS sheets bonded together. Before bonding, a rectangular opening (24 × 18 mm^2^) was cut using a cutter plotter, used to mould the hydrogel samples.

### HA-am hydrogel sample preparation

**Fig. 2:**
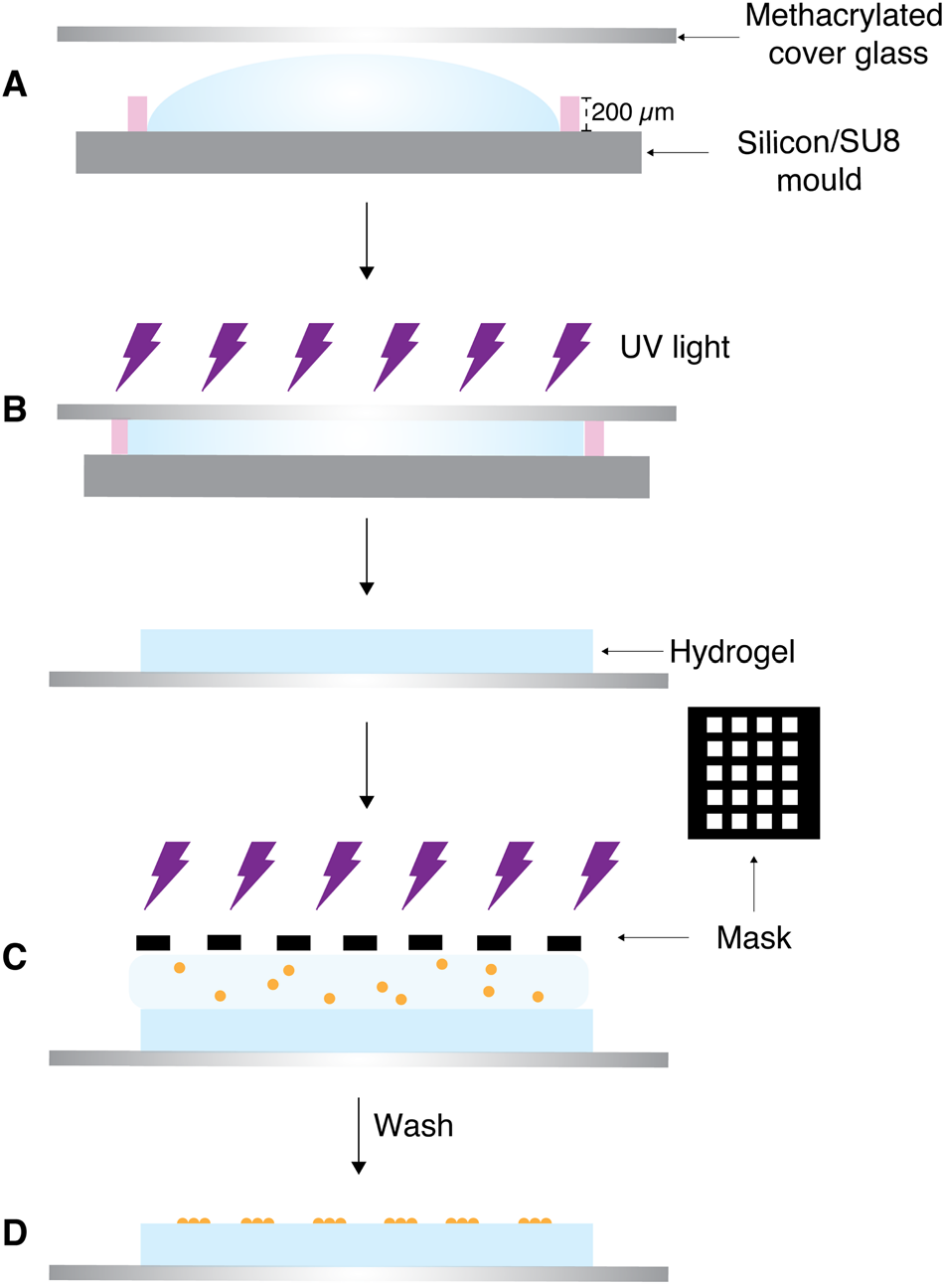
Schematic of HA-am hydrogel formation and patterning with RGD peptides. **A** Hydrogel precursor solution (HA-am 2% (w/v)) and photo-initiator (Irgacure 2959 0.4% in DPBS) was placed in the Si/SU8 mould and covered by a methacrylate-functionalized cover glass. **B** UV exposure. **C** RGD-peptide solution was pipetted on top of the gel, covered with a photomask and then exposed to UV light. **D** Unreacted peptides were removed by a washing step with DPBS

Irgacure 2959 photo-initiator was dissolved in DPBS at a concentration of 0.4% (w/v) and HA-am was dissolved in the initiator solution at a concentration of 2% (w/v). 12 µL of the solution was placed in the mould and covered by a methacrylate-functionalized cover glass, prepared following a previously published protocol [25]. The system was then irradiated with UV light to cross-link the HA-am derivative. Figure 2A, B shows a schematic of the fabrication process.

### Varying the UV light exposure dosage

For the UV light exposure, a UV source (UV LED 356 nm Curing lamp, Tao Yuan Electron) with a constant power of 100 W, mounted onto a 3D-printed holder 10 cm in height was used. A calibration curve of the resulting UV intensity was provided by the manufacturer (0.24 W/cm^2^ at 10 cm distance). For cross-linking and functionalization, the UV dose was varied by adjusting the exposure time at second intervals.

### Mechanical characterisation

Hydrogel samples were prepared using one of the PDMS moulds (24 × 18 × 1.5 mm^3^) described above bonded onto a glass bottom. The hydrogel precursor was added in the mould and covered by another glass coverslip. The hydrogel precursor was exposed to varying UV doses (1.4–8.2 J/cm^2^). Afterwards, the mould was disassembled and the hydrogel was left in supplemented cell media overnight. Three discs (8 mm diameter) were punched from the hydrogel and frequency sweep experiments were carried out on a Discovery HR-2 rheometer (TA Instruments) with a 8 mm Peltier steel parallel plate at 37 °C. A Poisons ratio of 0.5 was assumed [26] and the Young’s modulus (*E*) was calculated using the approximation *E* = *G*′*3, where *G*′ is the shear modulus at a frequency of 1 Hz. The value was chosen at 1 Hz as this is in the linear viscoelastic region.

### Patterning of HA-am hydrogel films with RGD peptides

After hydrogel cross-linking, 10 µL of a 0.5 mM RGD-peptide solution was pipetted on top of the gel, covered with a photomask and then exposed to UV light. Subsequently, unreacted peptides were removed by a washing step with DPBS for 2 h. Figure 2C, D shows a schematic of the fabrication process. The samples were left in supplemented media overnight inside an incubator (37 °C, 5% CO_2_). Different UV light intensities (0.24–6.9 J/cm^2^) were evaluated to identify the minimum UV intensity required to ensure sufficient binding of RGD peptide and good pattern resolution.

Previously, when RGD-peptide sequences have been used to control cell adhesion, a longer sequence (GCGYGRGDSPG) has typically been used on HA-based biomaterials [10, 13–15, 17, 18, 27, 28], whereas a shorter peptide sequence (RDGSC) has typically been used on synthetic hydrogel materials [29–32]. In this study, we investigated cell adhesion using both the longer and the shorter sequences. For visualisation of the patterns, we used fluorescently labelled versions of both peptide sequences, leaving a total of four different peptides used in the study.

### Cell culture on HA-am-RGD hydrogels

Mouse brain endothelial cells (bEnd.3) were cultured in DMEM/Nutrient Glutamax supplemented with 10% FBS and 1% Penicillin Streptomycin. Cells were maintained at 37 °C in 5% CO_2_. bEnd.3 cells, passage between 25 and 35, were seeded on the HA-am hydrogels (patterned or non-patterned) at a density of 25,000 cells/cm^2^ in supplemented media. Cell viability was assessed by Live/Dead staining assay on days 1 and 7 using a solution containing 1000X Calcein AM, 1000X Propidium Iodide and 1000X Hoechst in MEM medium (without phenol red and without FBS). Cells were incubated with the solution for 10 min and subsequently washed with MEM medium and imaged immediately after.

### Visualisation

Images were acquired using either a laser confocal microscope (Leica SP8) equipped with a photon multiplier detector and a hybrid detector taking z-stacks using a 10X objective or with an inverted microscope (Olympus IX73) equipped with Orca-Flash 4.0 LT digital CMOS camera.

## Results

### Simplified HA hydrogel preparation and patterning

**Fig. 3:**
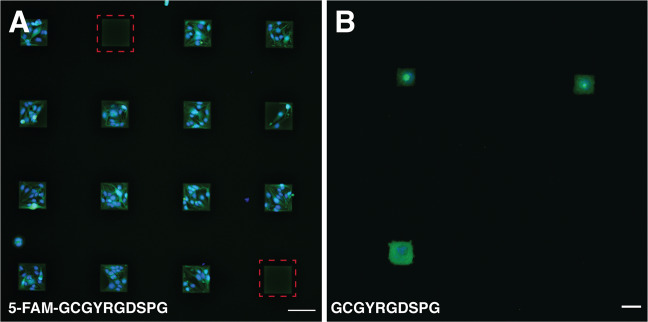
Fluorescent images showing bEnd.3 cells growing selectively on square peptide patterns on the hydrogel surface on day 1. **A** 100 × 100 µm^2^ 5FAM-GCGYRGDSPG patterns. Square green patterns of the RGD are visible, where cells did not adhere (marked in red dashed lines). Scale bar 100 µm. **B** 25 × 25 µm^2^ square GCGYRGDSPG patterns. Substantially lower cell adhesion was observed on the smaller patterns. Scale bar 25 µm. Viable cells within the patterns are stained in green and dead cells (non-observed) are stained in red. Cell nuclei stained in blue (colour figure online)

We demonstrate that the same chemical pathway, i.e., radical addition reaction, can be used for both cross-linking of HA-am precursor solution and spatially controlled functionalization of the obtained hydrogel with the cell-adhesion RGD peptide in square areas with dimensions ranging from 100 × 100 µm^2^ down to 25 × 25 µm^2^, Fig. 3. Hydrogel areas of 200 × 200 µm^2^ could also be patterned with RGD peptide, Figs. 5 and 7. Squares as small as 10 × 10 µm^2^ could be functionalized with RGD peptide, Fig. 5, but for these areas no cell adhesion was observed. We conclude that cross-linking of HA-am derivative at a dose of 4.6 J/cm^2^ was most suitable as it results in a hydrogel scaffold that was easy to handle and proved to be stable for long periods of time (~30 days) under cell culture conditions. The optimal UV dose for patterning of the hydrogel was identified to be 1.9 J/cm^2^ since it showed high pattern fidelity in combination with selective cell adhesion to the patterned areas. Below follow details on the systematic characterisation of the hydrogel preparation and functionalization.

### Hydrogel formation and mechanical characterisation

**Fig. 4:**
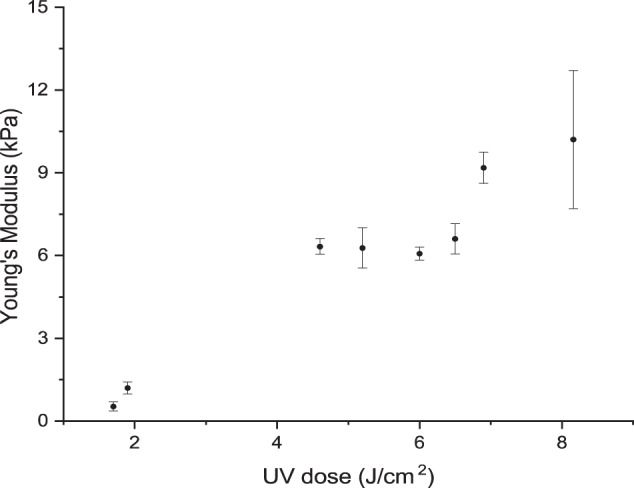
Young’s Modulus of the bulk hydrogel cross-linked at different UV doses. Error bars represent standard deviation from *n* = 3

The minimum UV dose required to form a cross-linked hydrogel was determined to be 1.7 J/cm^2^ resulting in a hydrogel with a Young’s modulus (*E*) of 0.53 ± 0.16 kPa. Increasing the UV dose resulted in an increase in the *E* of the hydrogel, as expected due to the higher degree of cross-linking, Fig. 4.

### Chemically patterned HA-am hydrogel samples

**Fig. 5:**
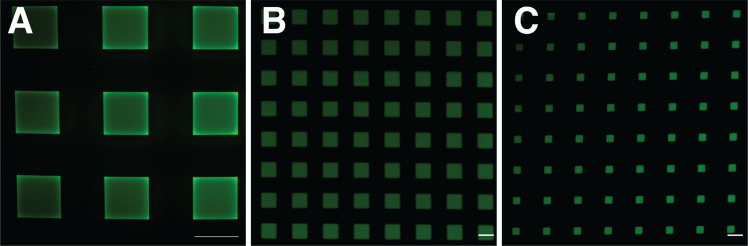
Square micropatterns of 5FAM-GCGYRGDSPG-peptide on HA-am hydrogels could be achieved using different masks. **A** 200 × 200 µm^2^, 200 µm spacing. **B** 10 × 10 µm^2^, 10 µm spacing. **C** 5 × 5 µm^2^, 15 µm spacing. Scale bar 200 µm in (**A**) and 10 µm in (**B**, **C**)

Patterning of RGD peptides to HA-am hydrogels was confirmed by visualisation of a pattern formed by the fluorescently labelled peptide, Fig. 5. Here, the hydrogel was cross-linked at a constant UV dose of 4.6 J/cm^2^ and the RGD peptides were linked using a UV dose of 1.9 J/cm^2^. Good resolution was obtained for squares down to 10 × 10 µm^2^, whereas rounded corners were observed for the 5 × 5 µm^2^ squares.

**Fig. 6:**
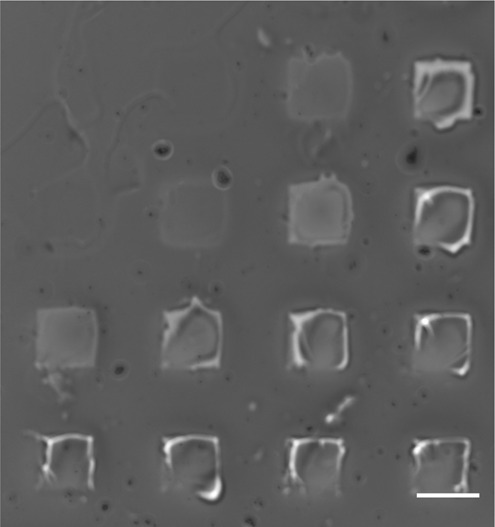
Bright field image of HA-am hydrogel exposed with a dose of 6.9 J/cm^2^ through a photomask with 200 × 200 µm^2^ openings. Topographical effects are observed in the areas exposed to the UV light. Scale bar 200 µm

It was observed that RGD peptides attached to the hydrogels with all UV exposure doses tested (0.24–6.9 J/cm^2^). However, at the lower doses, a lower florescence intensity was observed, indicating that less RGD peptides were attached to the hydrogel. As the same pathway is used for both the cross-linking of the hydrogel and the RGD functionalization, it means that the extra UV dose to attach the RGD peptides will also increase the cross-linking density in the exposed areas. It is therefore important to identify the maximum UV exposure dosage that the hydrogel can receive before topographical changes were observed in the hydrogel, resulting from extreme cross-linking of the hydrogel precursor solution. Such indentations could affect the cell adhesion and proliferation and in our work, we observed these defects when the HA-am hydrogel was exposed to 6.9 J/cm^2^, Fig. 6. Hence, we decided to use an exposure dosage of 1.9 J/cm^2^ for patterning the RGD peptides in this study to avoid any indentations in the gel.

### Cell adhesion on functionalized hydrogels

To investigate if the RGD-peptide patterns supported cell adhesion and proliferation, brain endothelial cells (bEnd.3) were seeded on the selectively functionalized hydrogels. For this purpose, HA-am hydrogels were initially cross-linked at a constant UV dose of 4.6 J/cm^2^, while the UV dose for RGD patterning was varied (0.24–4.6 J/cm^2^). bEnd.3 cells adhered to RGD patterns prepared using a UV dose of 1.9, 2.3 or 4.6 J/cm^2^, but there was no cell adherence to samples prepared using a UV dose of 0.24, 0.95 or 1.7 J/cm^2^, Table 1. When RGD peptides were patterned using 4.6 J/cm^2^, cells were observed to adhere in-between the patterns, due to over exposure, resulting in RGD adherence to the HA-am hydrogel outside the pattern areas.

**Table 1:** The cell response varied for different UV dosages tested for the RGD functionalization. The HA-am hydrogels were formed using a constant UV dose of 4.6 J/cm^2^

UV dose for RGD functionalization (J/cm^2^)	Young’s modulus (kPa)	Cell response
0.24		No cell adhesion
0.96		No cell adhesion
1.7	0.53 ± 0.16	No cell adhesion
1.9	1.12 ± 0.22	Cell adhesion
4.6	6.32 ± 0.28	Cell adhesion, cells crossing between the patterns

As we used the same functional groups on the HA-am for forming the gel and attaching the RGD peptide via radical addition reaction, we also wanted to identify the maximum UV exposure dosage the HA scaffold could be subject to during cross-linking, while still having sufficient number of Am groups left to react with the RGD peptides to support cell adhesion. This was evaluated by varying the UV dose to cross-link the hydrogel (4.0–10.6 J/cm^2^), while keeping the UV dose for the RGD patterning constant (1.9 J/cm^2^). In our system using HA-am with 14% functionalization, the maximum UV exposure dose to form the gel and still allow for RGD patterning and cell adhesion was found to be 8.16 J/cm^2^, Table 2. At UV dosages from 10.6 J/cm^2^ and above, cells would not adhere to the patterns. This indicates that there were not enough Am groups available to attach sufficient RGD peptides to permit cell adhesion to the hydrogels.

**Table 2:** When using different UV dosages to form the hydrogel, the cell response varied. The HA-am hydrogels were funcitonalized with RGD using a constant UV dose of 1.9 J/cm^2^

UV dose to form the hydrogel (J/cm^2^)	Young’s modulus (kPa)	Cell response
4.0		Cell adhesion
4.6	6.32 ± 0.28	Cell adhesion
6.9	9.18 ± 0.57	Cell adhesion
8.2	10.20 ± 2.50	Cell adhesion
10.6		No cell adhesion

### Role of peptide sequence on cell adhesion

To investigate the role of the RGD peptide related to the cell adhesion on the HA hydrogel, we prepared hydrogel samples initially cross-linked at 4.6 J/cm^2^ and used the four different peptide sequences to form patterns with a constant UV dose of 1.9 J/cm^2^. The results are summarised in Table 3.

**Table 3:** Cell adhesion and proliferation on different RGD-peptide sequences

Peptide sequence	Fluorescent	Cell adhesion
RGDSC	No	No
5FAM-RGDSC	Yes	Yes
GCGYRGDSPG	No	Yes
5FAM-GCGYRGDSPG	Yes	Yes

**Fig. 7:**
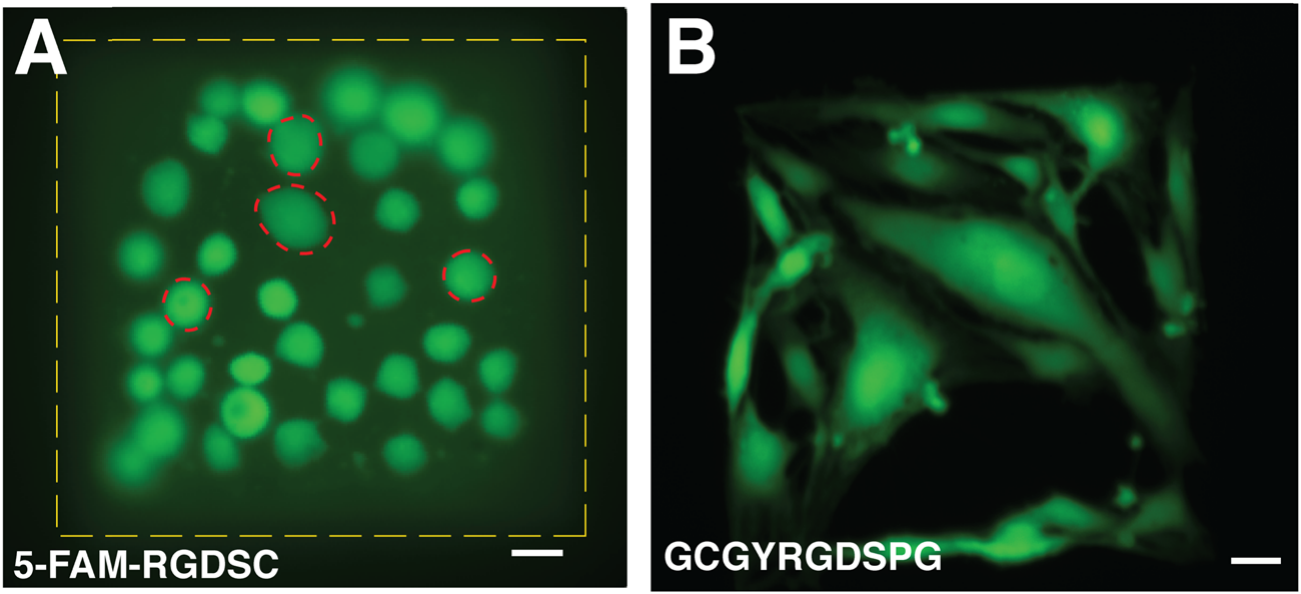
bEnd.3 morphology on different peptide sequences. **A** Fluorescent image (day 7) showing bEnd.3 cells growing selectively on the peptide functionalized area (200 × 200 µm^2^), marked with a yellow dashed line. The cells remain alive but do not elongate (marked with red dashed line). **B** bEnd.3 cells show an elongated morphology on the pattern (200 × 200 µm^2^) formed by the longer peptide sequence already at day 1. Scale bar is 20 µm (colour figure online)

For the shorter peptide sequence, it was observed that the bEnd.3 cells did not adhere to the non-fluorescent CRGDS patterns, whereas the cells did adhere to the fluorescently labelled 5FAM-CRGDS patterns. However, the cells showed a rounded morphology and did not spread on the pattern, even after 7 days in culture, Fig. 7. This indicates that 5FAM-RGDSC peptide supported cell viability but that they do not provide the optimal substrate for endothelial cell adhesion. For the longer peptides, bEnd.3 cells adhered to both the fluorescently labelled RGD-peptide sequences, 5FAM-GCGYRGDSPG, and the non-labelled sequence, GCGYRGDSPG, and showed a healthy phenotype [33] with an elongated morphology characteristic of endothelial cells on both patterns, Fig. 7.

## Discussion

In this work, we present a straightforward and simplified approach to fabricate 200 µm thick HA hydrogels, where the cell adhesion of brain endothelial cells can be controlled on the micrometre scale. The fabrication of these hydrogel-based cell culture substrates relies on two sequential and short UV exposure steps. An advantage of this simplified method, using UV for both the formation of the hydrogel and the patterning, is that the stiffness of the hydrogel can be fine-tuned independently from the polymer degree of functionalization, which is not the case when, e.g., Michael-type addition reactions are used. In such reaction schemes, the hydrogel will cross-link until all functional groups (or all cross-linker) are consumed and the stiffness of the resulting hydrogel is thus determined at the start of the process, by the degree of functionalization of the precursor material or the initial cross-linker concentration. Our approach permits fine-tuning of the stiffness of the resulting hydrogel after precursor synthesis and tailoring for the desired application during the fabrication of the scaffold. In our system, we can adjust the stiffness of the material by carefully choosing the appropriate hydrogel cross-linking UV dose and the patterning dose to obtain the desired stiffness that the cells will experience. This behaviour has also been shown in similar systems where HA methacrylate was exposed to varying UV exposure times (0–90 s) generating a mechanical gradient throughout the hydrogel from ~3 to 100 kPa [12]. In this work, where the adhesion of brain microvasculature cells were studied, we identified a UV dose of 4.6 J/cm^2^ for forming the gel followed by 1.9 J/cm^2^ UV exposure for the patterning RGD peptides, as the best settings. This resulted in an initial hydrogel stiffness of 6.3 ± 0.3 kPa, which was then increased to 6.6 ± 0.6 kPa after the RGD patterning. The resulting gel showed optimal properties as it permitted easy handling and stability over a relevant time span for cell culture. Lower exposure dose for peptide patterning resulted in impaired cell adhesion as a result of too low peptide binding concentration. A higher exposure dose, on the other hand, resulted in over-exposed gels as seen by cells adhering outside the specified patterns. Moreover, we could achieve high-fidelity patterns down to 10 × 10 µm^2^. In standard UV lithography where patterns are transferred onto a solid substrate, one can usually obtain high-resolution patterns down to 1 × 1 µm^2^ but due to the soft nature of the hydrogel, we could not perform the exposure under hard contact conditions, thus reducing the resolution. A pattern resolution of 10 × 10 µm^2^ will permit future modelling of the brain microvasculature, as brain capillaries are 7–10 μm in diameter [34].

In addition, we explored if different RGD sequences would affect the adhesion and spreading of the brain endothelial cells in our scaffold as it has been shown that the structural conformation of the RGD-peptide sequence affects cell-adhesion affinity [35]. Moreover, it has been reported that cell adhesion to RGD peptides on different surfaces depends on the distance between the peptide and the surface, where longer RGD peptides have shown improved cell-adhesion properties [36–39]. This might be an explanation to why we only observe cell adherence on the longer peptide but not on the patterns formed by the shorter RGD peptides. Interestingly, cells did not adhere to the shorter non-fluorescent RGD peptide, but could still adhere and remain viable on the shorter fluorescent counterpart, while failing to elongate and instead displaying a rounded morphology. This is probably due to an inability to establish specific interactions between the integrin receptors on the cell surface and the RGD peptide. We believe that this platform could have a major applicability in the tissue engineering field, for the development of organs-on-chip models as well as in high throughput applications in the future.

## Conclusion

In the present study we demonstrated a simplified method where HA-am derivative can participate in sequential photo-initiated radical addition reactions to form hydrogel films with spatially defined cell adhesive peptides. The functionalization of HA hydrogel with RGD peptides was possible due to UV light-triggered radical thiol–ene addition of the thiol group of the peptide to the Am groups that remained unreacted after the photo-cross-linking step. Using UV lithography, we were able to generate the RGD patterns in spatially defined areas of the hydrogel film, as small as 10 × 10 µm^2^. In addition, we showed that bEnd.3 cells selectively adhere to and proliferate on the longer GCGYRGDSPG-peptide patterns whereas the shorter CRGDS-peptide patterns only support adhesion. This demonstrates that HA-am derivative is a promising material for fabrication of in vitro models with in situ HA hydrogel formation.
